# Effects of school time on sleep duration and sleepiness in adolescents

**DOI:** 10.1371/journal.pone.0203318

**Published:** 2018-09-26

**Authors:** Seonkyeong Rhie, Kyu Young Chae

**Affiliations:** Department of Pediatrics, CHA Bundang Medical Center, CHA University, Seongnam, Republic of Korea; University of Lübeck, GERMANY

## Abstract

Delaying the time of start of school allows for longer sleep duration, better mood, and better school performance. In South Korea, a campaign was launched in 2014 to delay the school start time to 9 a.m. We analyzed the campaign’s effects on adolescents’ total sleep duration, sleepiness (presented as weekend catch-up sleep), emotions, and school performance. Based on data from 2013, changes of sleep patterns, emotions, and academic achievement in adolescents were evaluated using the 2012–2016 Korea Youth Risk Behavior Web-based survey from two educational districts: Gyeonggi (fully participated in the delayed school start time campaign; intervention group) and Daegu/Gyeongbuk/Ulsan (DGU, never participated; control group). The primary outcomes were sleep duration, time of sleep onset, and difference in sleep duration between weekdays and the weekend. Secondary outcomes were the proportional changes of mood, stress, and school performance. The sleep duration of students in the intervention group temporarily increased in 2015. However, because there was a simultaneous delay in time of sleep onset, sleep duration returned to pre-campaign levels in 2016. Although sleep duration did not increase, weekend catch-up sleep decreased by approximately 19 minutes for students in the intervention group. Meanwhile, in the control group sleep duration tended to decrease over the same period. The impact of the campaign on students' emotions and school performance could not be confirmed. This study demonstrated that delaying the school start time to 9 a.m. reduced duration of weekend catch-up sleep with a transient increase in sleep duration in adolescents.

## Introduction

Sleep is essential for sustaining a vibrant and healthy life, and it is a key contributor to emotional stability and learning abilities, such as memory, cognition, and executive function [1, 2]. Sleep duration is influenced by sleep onset and wake-up times. In particular, a physiologically-delayed sleep phase [3, 4] in adolescents shortens overall sleep duration [5, 6] due to a fixed early school start time. Several studies [7] have concluded that a late school start time increases sleep duration and improves school performance. Owens et al. [8] showed in a study of 357 Rhode Island high school students that a modest delay in school start time from 8:00 to 8:30 a.m. was associated with significant improvements in adolescent alertness, mood, and health. Lufi et al. [9] presented similar results from a short-term study. A 25–60-min delay in school start time increased total sleep time by 25–77 min [10]. However, these studies were limited by poorly defined control groups, small sample sizes, and especially, short observation periods [11].

Based on these results, several local offices of education in Korea implemented a delayed school start time. These campaigns began between fall and winter 2014. Some municipalities delayed school start times, some did not, and some allowed the principal of each school to determine whether to enforce it, as there were many pros and cons debated before the campaign. The main objection was that it would simply delay the students' daily activities, and that due to a competitive environment, students would be taking private lessons until late at night. However, a survey by the Gyeonggi Institute of Education [12] involving 13,000 students showed improved mood and wakefulness immediately after the campaign was implemented. This long-term observational study sought to confirm the effects of delayed school start time on sleep duration, school performance, mood, and sleepiness through the use of a population-based survey with a control group.

## Materials and methods

### Data selection

In this study, students from educational districts in Gyeonggi, Daegu, Gyeongbuk, and Ulsan (DGU) were recruited. Of the 17 educational districts in South Korea, 6 (Seoul, Gyeonggi, Incheon, Gangwon, Jeonbuk, and Jeju) delayed the school start time until 9:00 a.m., 8 (Chungbuk, Chungnam, Sejong, Daejeon, Gwangju, Jeonnam, Gyeongnam, and Busan) were delayed at the discretion of the principal, and the remaining 3 (Daegu, Gyeongbuk, and Ulsan) did not change the school start time. Among the 14 educational districts that fully or partially enforced the 9:00 a.m. school start time, the actual enforcement rate varied; Gyeonggi and Gangwon fully participated, and Seoul partially participated [13, 14]. However, Gangwon was excluded from the study due to a smaller sample size (3%) compared with that of other districts. We enrolled students from the Gyeonggi districts (intervention group) and from the DGU districts (control group), where no schools participated in the delayed start campaign. The campaign onset was from September 2014 to March 2015; we used the Korea Youth Risk Behavior Web-based Survey (KYRBS) data for 2012, 2013, 2015, and 2016, but not for 2014 (S1 Table).

Before 2014, most middle school students in the country began classes at 8:00–8:10 a.m., and high school students at 7:30–7:40 a.m. Before the campaign, most middle school students in the intervention group began class at 8:00–8:30 a.m. (96%), and 50–55% of high school students began school by 7:40–8:00 a.m. [12]. In October 2014, after initiation of the delayed school start campaign, 98.8% of middle school and 81.6% of high school began at 9:00 a.m. As a result, the mean school start time was delayed by approximately 30 minutes to 1 hour for middle school students and by 1–1.5 hours for high school students. Over this period, the school start time in the DGU districts continued to be 7:30–8:00 a.m.

### Data source: The Korea Youth Risk Behavior Web-based Survey (KYRBS)

The KYRBS, developed by the Centers for Disease Control and Prevention in South Korea, has been a regular online survey since 2005 to assess health risk behaviors in adolescents every year. The number of online research subjects is determined by the ratio of area and age, and the results are anonymous. Survey data can be used with permission. The questionnaires consist of 125 items in 15 areas, such as use of alcohol or drugs, physical activity, stress, smoking, sleep habits, and others. Data are stratified by district and age from 800 sampled schools. The survey period for this study was between June 1 and June 30 for all of 2012–2016.

### Survey item selection

Data between 2012 and 2016, excluding data for 2014, in which the campaign began, were collected from students in the intervention and control groups. The sleep onset time, wake-up time, and sleep duration on the weekdays and weekend for the last 7 days before survey results were collected, and sleep duration difference between weekdays and the weekend were calculated. Depressive mood and suicidal ideation in the past 12 months, stress level, fatigue recovery from sleep for the last 7 days, school performance as grade point average, grade, sex, and age were also investigated.

### Exclusion criteria

Data were excluded if there was an error in the representation of the sleeping time. In other words, students were excluded if the reported sleep onset time started between 9:00 a.m. and noon or if the total sleep time was <3 hours or >16 hours. In addition, third-year high school students were excluded because they had the highest possibility of an irregular sleep pattern because of examinations.

### Data calculations

Because sleepiness scores were not measured directly, they were calculated as the difference between weekday and weekend sleep durations, also referred to as weekend catch-up sleep, which can be a proxy for sleepiness [15]. Thus, a greater difference of sleep duration meant students slept longer on the weekend. Since sleep time on the weekend was not measured in 2012, only information for 2013/2015/2016 was included in this survey.

### Statistical analysis

The KYRBS is a stratified survey. For this study, we analyzed the survey data using the complex sampling method proposed by the Korea Centers for Disease Control and Prevention (KCDC), with weighting and clustering. When comparing continuous variables in regression analyses and complex samples, the reference for identifying differences by year was based on 2013 data, just before the start of the campaign. To compare the mean sleep onset time, wake-up time, and sleep duration, a general linear model in the complex-samples method was used. To compare the frequencies of mood, school performance, stress, and suicidal ideation, a logistic regression analysis using complex samples was used. Results are presented as estimated means and 95% confidence intervals (CIs). Two-sided tests with p-values < 0.05 were considered statistically significant. All statistical procedures were performed using the Statistical Package for the Social Sciences 18.0 for Windows (SPSS, IBM, Armonk, NY).

### Ethics

The institutional review board of the CHA Bundang Medical Center approved the study protocol (IRB number CHA 2016-12-054).

## Results

### Sample characteristics

Overall attendance of KYRBS included 66,000–74,000 students, and the participation rate was 96.2–96.4% each year (S1 Table). After excluding outliers, errors in weekday sleep hours, and high school seniors, 70.9% of students in 2012, 72.1% in 2013, 71.9% in 2015, and 72.5% in 2016 were included. The number of students included in this study was 42,517 from the intervention group and 28,287 from the control group. These students represented an estimated 2,488,642 and 1,281,123 students, respectively (Table 1). Students from the intervention group accounted for 24.4–25.6% and students from the control group accounted for 12.7–13.2% of the nationwide population. Male students accounted for 51.2–51.6% of the total population studied. The number of students per grade was evenly distributed.

**Table 1:** Survey population in two districts.

		2012	2013	2015	2016
		Raw number	Raw number	Raw number	Raw number
		Represented (%)	Represented (%)	Represented (%)	Represented (%)
District	intervention	11062	11101	10223	10131
		645194 (24.4%)	661302 (25%)	599727 (25.2%)	582419 (25.6%)
	Control	7608	7566	6771	6342
		347904 (13.2%)	340641 (12.9%)	304091 (12.8%)	288487 (12.7%)
Grade	7^th^	10188	10236	8997	8864
		487697 (18.4%)	503575 (19.1%)	383820 (16.1%)	396000 (17.4%)
	8^th^	10240	10263	9682	8937
		502721 (19.0%)	499695 (18.9%)	444213 (18.6%)	390654 (17.2%)
	9^th^	10588	10534	10338	9654
		537676 (20.3%)	523183 (19.8%)	506302 (21.2%)	449179 (19.8%)
	10^th^	10763	10639	9865	10101
		553670 (20.9%)	558377 (21.1%)	516872 (21.7%)	522520 (23.0%)
	11^th^	10828	10582	10028	9948
		561816 (21.3%)	555598 (21.0%)	531956 (22.3%)	513903 (22.6%)
Sex	male	26796	26136	25080	24178
		1363393 (51.6%)	1363634 (51.6%)	1226542 (51.5%)	1163160 (51.2%)

Raw numbers and represented numbers of students (proportion of nationwide population)

### Sleep onset time and duration

The sleep duration on weekdays in intervention group was 6 h 34 min (95% CI, 6 h 29 min–6 h 40 min) in 2012, 6 h 39 min (6 h 33 min–6 h 45 min) in 2013, 6 h 49 min (6 h 43 min–6 h 55 min) in 2015, and 6 h 39 min (6 h 34 min–6 h 45 min) in 2016 (Table 2). There was no significant difference in the sleep onset time between 2012 and 2013 in the intervention group, but in 2015 and 2016 it was significantly delayed compared to that in 2013, with a difference of 15–26 min. The sleep onset time of adolescents in the control group was similar in 2012, 2013, and 2015, but slightly delayed by 5 minutes in 2016. The wake-up time in the intervention group was delayed by about 30 minutes in 2015 and 2016 compared to that in 2013, whereas in the control group, it did not differ over time. As a result, the total sleep duration temporarily increased in 2015 by approximately 10 minutes, but after that, the difference disappeared in 2016 in the intervention group by longitudinal study However, sleep duration in the intervention group was significantly longer than the control after campaign.

**Table 2:** Sleep onset, wake-up time, and sleep duration.

		2012	2013	2015	2016	p-value		
						2012	2015	2016
Sleep onset, time, mean (95%CI)							
intervention	00:13 (00:09–00:17)	00:07 (00:03–00:11)	00:23 (00:18–00:28)*	00:34 (00:29–00:39)*	.053	.000	.000
control	00:14 (00:07–00:20)	00:11 (00:05–00:17)	00:17 (00:10–00:23)*	00:23 (00:16–00:29)*	.590	.226	.016
Wake up hours, time, mean (95% CI)							
intervention	6:48 (6:46–6:51)	6:47 (6:44–6:50)	7:13 (7:10–7:15)*	7:14 (7:11–7:16)*	.509	.000	.000
control	6:49 (6:47–6:52)	6:48 (6:45–6:51)	6:48 (6:45–6:51)*	6:49 (6:46–6:51)*	.470	.993	.721
Sleep duration, hours, mean (95% CI)							
intervention	06:34 (06:29–06:40)	06:39 (06:33–06:45)	06:49 (06:43–06:55)*	06:39 (06:34–06:45)*	.273	.034	.978
control	06:35 (06:27–06:43)	06:36 (06:28–06:44)	06:31 (06:22–06:39)*	06:25 (06:17–06:34)*	.863	.368	.083

Time (95% confidence interval), p-value from 2012 to 2016: compared with 2013,

* p-value < 0.05 between intervention and control group; complex samples general linear model

### Difference between sleep duration on weekdays compared to the weekend

The differences between the weekday-weekend sleep duration were not significant in the two groups in 2013 (109–110 min). After adjusting for the students’ sex and grade (Table 3), before the campaign, there was a weekend catch-up sleep for students in the two areas of 144–149 min, and these did not significantly differ by region. After the campaign, students’ weekend catch-up sleep in the intervention group significantly decreased by approximately 19 min compared to that in 2013 (95% CI 15.0–23.2 in 2015, 14.4–22.8 in 2016), where the control group showed no change.

**Table 3:** Differences in sleep duration between weekday and weekend.

	Year	Estimate	95% CI		
			Lower	Upper	Sig.
Intervention group	2013	148.767	144.121	153.413	
2015*	-19.106	-23.245	-14.968	.000
2016^¶^	-18.618	-22.804	-14.432	.000
Control group	2013	148.803	142.918	154.687	
			.	.	.
	2015*	-1.718	-6.831	3.395	.510
	2016^¶^	-2.900	-8.784	2.984	.334

Adjusted for grade and sex, *^¶^ p-value < 0.05 between the intervention and the control group

### Emotions and school performance

Subjective health perceptions improved in 2015 and 2016, compared to those in 2013, with no significant differences by group (“very healthy” and “healthy”) (Table 4). Subjective happiness also improved by year (“very happy” and “happy”), regardless of group. Feeling stressed (“felt very much stressed” and “felt a lot stressed”) decreased by year in both groups. The degree of fatigue recovered from sleep for the past 7 days in the control group was rated as “very sufficient”, and “sufficient” temporarily improved and returned to that of the pre-campaign, while the ratings in the control group did not change by year. Depression during the past 12 months and suicidal thoughts and plans for past 12 months decreased in both groups. With respect to school performance presented as grade point average, highs and moderate grade point averages increased year by year, regardless of the location.

**Table 4:** Changes in mood and performance based on the year.

	2012	2013	2015	2016	*P*-value^*^		
					2012	2015	2016
Subjective Health Perception: Very health & Healthy							
intervention	67.9% (66.7–69.2)	69.3% (68.1–70.6)	73.3% (72.1–74.4)	71.3% (70.0–72.5)	.127	.000	.033
control	67.7% (66.0–69.4)	70.2% (68.5–71.8)	74.5% (72.9–75.9)	73.3% (71.4–75.0)	.039	.000	.015
Subjective happiness: Very happy & Happy							
intervention	57.3% (56.0–58.5)	58.4% (57.1–59.7)	65.6% (64.1–67.0)	66.6% (65.4–67.8)	.226	.000	.000
control	56.2% (54.5–57.8)	57.6% (55.7–59.5)	67.0% (65.4–68.6)	68.9% (66.9–70.7)	.271	.000	.000
Feeding stressed: felt very much & a lot stressed							
intervention	41.2% (39.9–42.4)*	41.4% (40.1–42.8)*	33.7% (32.4–35.0)	37.7% (36.2–39.2)*	.801	.000	.000
control	38.8% (37.0–40.7)*	38.5% (36.6–40.3)*	31.9% (30.3–33.6)	32.9% (31.1–34.7)*	.784	.000	.000
Fatigue recovered from sleep for the last 7 days: Very sufficient and Sufficient							
intervention	29.2% (27.7–30.7)	26.4% (25.1–27.7)	31.1% (29.4–32.9)*	26.7% (25.2–28.1)	.006	.000	.756
control	28% (25.6–30.5)	24.4% (22.2–26.6)	26.9% (24.7–29.2)*	25.6% (23.1–28.3)	.030	.114	.470
Depression during last 12 months: Yes							
intervention	29.3% (28.3–30.4)*	30.0% (28.8–31.2)*	22.4% (21.4–23.5)*	25.5% (24.5–26.6)*	.425	.000	.000
control	27.2% (25.7–28.6)*	27.1% (25.6–28.6)*	20.2% (19.0–21.4)*	20.8% (19.5–22.1)*	.927	.000	.000
Suicidal thinking during last 12 months: Yes							
intervention	17.9% (17.1–18.8)	16.5% (15.6–17.4)*	11.4% (10.7–12.2)*	12.5% (11.8–13.3)*	.022	.000	.000
control	16.7% (15.5–18.0)	14.4% (13.4–15.6)*	9.7% (8.8–10.7)*	9.6% (8.7–10.5)*	.008	.000	.000
Plan for suicide during last 12 months: Yes							
intervention	5.9% (5.4–6.5)	5.7% (5.2–6.2)*	3.4% (3.0–3.8)	4.0% (3.6–4.5)*	.519	.000	.000
control	5.3% (4.7–6.0)	4.4% (3.9–4.8)*	2.9% (2.4–3.5)	3.0% (2.5–3.5)*	.021	.000	.000
School performance as GPA: high and mid-high							
intervention	34.3% (33.1–35.6)*	33.9% (32.6–35.2)*	38.4% (37.0–39.9)	37.8% (36.5–39.1)	.629	.000	.000
control	39.8% (37.8–41.9)*	36.7% (34.8–38.6)*	40.6% (38.6–42.6)	39.4% (37.6–41.4)	.026	.005	.037

Estimated rate (99% CI), ^*^P-value from 2012 to 2016 compared with 2013, GPA: grade point average,

* p-value < 0.05 between intervention and control group; complex samples logistic regression

## Discussion

This study demonstrates that delaying the school start time transiently increased the sleep duration of adolescents without a change over the long term. Recovery from fatigue by sleep was only slightly and transiently associated with the campaign. Self-reported school performance of the intervention group was more improved than the control. However, this study could not find the effects of the campaign on emotions such as depression and suicidal thinking/plan. Most importantly, this study shows that sleepiness, represented as the difference in sleep duration between weekdays and the weekend, was reduced after delaying the school start time [16]. This was a 5-year prospective case-controlled cohort study based on the KYRBS data. Although previous studies [8, 9, 17] also investigated delaying school start times in adolescents, their sample sizes were too small or their control groups were not clearly defined. In addition, most relevant studies [8, 9] had a short observational duration following the delayed school start time implementation. The present study is also meaningful because, to our knowledge, there have been few studies on adolescents with severe sleep restriction in Asia, where most of the middle or high school students attend private institutions lasting until late at night. Similar to previous studies [18, 19], we found that Korean students slept too little.

This study also shows that when school start time is delayed, sleep onset time and wake-up times of adolescents were also delayed, with a transient increase in sleep duration. Previous studies by Wolfson and Wahlstrom concluded that delaying school start time has significant benefits in terms of sleep duration [20, 21]. However, a review by Marx et al. recently concluded that the effect of the later school start times could not be determined due to limited, and generally low quality, evidence [11]. In Korea, opponents of the campaign also predicted that, although the school start times would be delayed, students would simply have delayed circadian cycles, due to their having the same schooling and private tutoring. However, in a short-term follow-up study [12] that examined the effects of the same campaign in Gyeonggi (the intervention group) with 3628 elementary school students, 4626 middle school students, and 4668 high school students, sleep duration was found to be significantly increased by 7 min in elementary schools, 17 min in middle schools and 31 min in high schools. This campaign seemed to work very well to improve the sleep duration for adolescents. The current study, however, demonstrated that sleep duration was transiently increased and returned to that of the pre-campaign. The reasons for the increased sleep duration not being sustained could be a result of too many extracurricular activities, homework, tutoring after school, and the use of electronic devices at bed time, especially since the use of social media may delay the diurnal cycle [22].

Although the campaign delayed the sleep phase without substantially increasing sleep duration, this study inferred that delaying the school start time significantly transiently decreased daytime sleepiness, which is reflected in the smaller differences of sleep duration between weekdays and the weekend. Insufficient sleep and sleep debt could cause a varied sleep duration. Klerman and Dijk [23] showed that sleep debts lead to longer sleep durations with extended sleep opportunities. Other studies [16, 24] also showed a correlation between sleepiness and attention deficits, and catch-up sleep on the weekend.

The reasons for a reduced weekend catch-up sleep without a substantial increase in sleep duration are not clear. It could be due to synchronization of the internal sleep phase with the social environment. Giannotti et al. [25] showed that many patients with weekend catch-up sleep have reduced sleepiness after matching the sleep phase with the environment. Therefore, this campaign could be considered as partially successful, especially for decreasing daytime sleepiness.

Unexpectedly, we found that mood and school performances in this study were improved in all districts, even where the campaign was not implemented. Therefore, not only did we conduct a thorough investigation to determine whether the contents of the KYRBS questionnaire in 2013–2016 were changed, but we also looked for potential factors that could have significantly affected the emotion of adolescents during the investigation periods. However, we found no changes in the KYRBS questionnaire or environmental changes that would improve emotion. Moreover, we could not identify factors that would improve the mood in all districts, regardless of implementation of the campaign. We were unable to correlate the improvements in mood and school performance with the delayed school start time based on this study. However, some previous studies reported that delayed school start times not only increased sleep duration but also emotional stability and academic efficiency. Edward et al. [7] reported that a one-hour delay of school start time improved reading and scores in mathematics. Owen et al. [8] reported that a 30-minute delay in school start time improved satisfaction scores associated with sleep, tiredness, and depression. Similarly, the study mentioned above [12], performed immediately after the campaign, found that the number of students who napped decreased, and that concentration in morning classes improved. However, this long term observational study could not clarify the effects of the campaign on emotion or academic performance.

### Limitations

In this study, daytime sleepiness and daytime napping could not be directly measured. Because sleepiness, defined by measures such as the pediatric daytime sleepiness scale [26], was not investigated, it was indirectly deduced through the difference in weekday and weekend sleep durations. In this study, recall data for sleep time was used instead of measuring it objectively. However, a previous study showed that recalled sleep time was not significantly different from actual sleep time [27]. School performance was also subjectively evaluated with self-reported data.

### Conclusions

This study demonstrated that delaying school start times could reduce duration of weekend catch-up sleep in adolescents. Our data showed that a 0.5–1.5-hour delay in the school start time led to a delayed sleep onset time and corresponding wake-up time, with a 10-minute increase in sleep duration that proved to be transient. However, the campaign reduced weekend catch-up sleep duration. It is highly possible that expansion of the campaign to a nationwide program could have a positive effect on adolescents with severely restricted sleep. Moreover, long-term follow-up investigations should be implemented around school start times, focusing on the effects of emotion and cognition, and providing social supports to ensure proper sleep for adolescents.

## Supporting information

S1 Table Students in each year based on district, academic year, and sex. (DOCX)
