# Supplementary material for: Effects of school time on sleep duration and sleepiness in adolescents
Source: PLoS One. 2018 Sep 26;13(9):e0203318. doi: 10.1371/journal.pone.0203318 (PMC6157849; doi:10.1371/journal.pone.0203318)
Supplement: S1 Table — (DOCX) [file pone.0203318.s001.docx]

**Supplementary Table 1. students in each year based on district, academic year, and sex**

|  |  | 2012 | | 2013 | | 2015 | | 2016 | |
| --- | --- | --- | --- | --- | --- | --- | --- | --- | --- |
|  |  | raw | represented (%) | raw | represented (%) | raw | represented (%) | raw | represented (%) |
| District | | | | |  |  |  |  |  |
|  | Gangwon | 2486 | 112003 (3.0%) | 2457 | 110636 (3.0%) | 2449 | 102167 (3.0%) | 2224 | 96779 (3.0%) |
|  | Gyeonggi | 15658 | 919818 (24.6%) | 15319 | 912199 (24.8%) | 14352 | 847641 (25.3%) | 13990 | 814765 (25.6%) |
|  | Gyeongnam | 4920 | 254976 (6.8%) | 4744 | 250227 (6.8%) | 4581 | 227123 (6.8%) | 4132 | 215294 (6.8%) |
|  | Gyeongbuk(G) | 3755 | 186412 (5.0%) | 3924 | 182858 (5.0%) | 3557 | 166476 (5.0%) | 3335 | 157632 (4.9%) |
|  | Gwangju | 3149 | 133773 (3.6%) | 3002 | 131749 (3.6%) | 2850 | 119749 (3.6%) | 2676 | 113126 (3.6%) |
|  | Daegu(D) | 4312 | 203178 (5.4%) | 4151 | 196824 (5.4%) | 3614 | 175186 (5.2%) | 3292 | 164816 (5.2%) |
|  | Daejeon | 2975 | 124228 (3.3%) | 2947 | 121917 (3.3%) | 2656 | 109900 (3.3%) | 2682 | 103876 (3.3%) |
|  | Busan | 4939 | 240452 (6.4%) | 4649 | 231034 (6.3%) | 4209 | 203332 (6.1%) | 3979 | 191051 (6.0%) |
|  | Seoul | 11373 | 656296 (17.5%) | 11165 | 634922 (17.3%) | 9710 | 568448 (17%) | 9567 | 536286 (16.8%) |
|  | Sejong | 0 | 0 (0%) | 0 | 0 (0%) | 935 | 10929 (0.3%) | 1012 | 13817 (0.4%) |
|  | Ulsan(U) | 2518 | 98190 (2.6%) | 2486 | 95123 (2.6%) | 2218 | 84622 (2.5%) | 2114 | 79351 (2.5%) |
|  | Incheon | 4302 | 209926 (5.6%) | 4140 | 204637 (5.6%) | 3847 | 186613 (5.6%) | 3724 | 177940 (5.6%) |
|  | Jeonnam | 2906 | 140625 (3.8%) | 2871 | 138460 (3.8%) | 2925 | 126072 (3.8%) | 2820 | 119043 (3.7%) |
|  | Jeonbuk | 3133 | 146352 (3.9%) | 3183 | 144539 (3.9%) | 2931 | 133185 (4.0%) | 3060 | 126570 (4.0%) |
|  | Jeju | 1743 | 47544 (1.3%) | 1560 | 47274 (1.3%) | 1597 | 44768 (1.3%) | 1478 | 43304 (1.4%) |
|  | Chungnam | 3264 | 153340 (4.1%) | 3112 | 153680 (4.2%) | 3095 | 137677 (4.1%) | 2990 | 131887 (4.1%) |
|  | Chungbuk | 2753 | 118622 (3.2%) | 2725 | 116495 (3.2%) | 2517 | 105875 (3.2%) | 2453 | 100175 (3.1%) |
| Grade (school year, academic year) | | | | | |  |  |  |  |
|  | 7^th^ | 12362 | 591363 (15.8%) | 12199 | 596448 (16.2%) | 10786 | 459562 (13.7%) | 10483 | 467407 (14.7%) |
|  | 8^th^ | 12384 | 609081 (16.3%) | 12113 | 588885 (16.0%) | 11442 | 522860 (15.6%) | 10517 | 457678 (14.4%) |
|  | 9^th^ | 12551 | 636047 (17.0%) | 12218 | 605804 (16.5%) | 12071 | 591650 (17.7%) | 11219 | 521162 (16.4%) |
|  | 10^th^ | 12451 | 639784 (17.1%) | 12028 | 629732 (17.1%) | 11122 | 582488 (17.4%) | 11355 | 587538 (18.4%) |
|  | 11^th^ | 12315 | 640940 (17.1%) | 11865 | 621612 (16.9%) | 11113 | 587943 (17.6%) | 11070 | 571174 (17.9%) |
|  | 12^th^ | 12123 | 628520 (16.8%) | 12012 | 630093 (17.2%) | 11509 | 605260 (18.1%) | 10884 | 580753 (18.2%) |
| Sex |  |  |  |  |  |  |  |  |  |
|  | Male | 38221 | 1966019 (52.5%) | 36655 | 1921266 (52.3%) | 35204 | 1746438 (52.1%) | 33803 | 1661456 (52.2%) |

Raw students, represented students, (proportion of nationwide population)
